# *Babesia ovis* secreted antigen-1 is a diagnostic marker during the active *Babesia ovis* infections in sheep

**DOI:** 10.3389/fcimb.2023.1238369

**Published:** 2023-08-16

**Authors:** Ferda Sevinc, Mo Zhou, Shinuo Cao, Onur Ceylan, Mehmet Can Ulucesme, Sezayi Ozubek, Munir Aktas, Xuenan Xuan

**Affiliations:** ^1^Department of Parasitology, Faculty of Veterinary Medicine, University of Selcuk, Konya, Türkiye; ^2^Jiangsu Key Laboratory for High-tech Research and Development of Veterinary Biopharmaceuticals, Jiangsu Agri-animal Husbandry Vocational College, Taizhou, China; ^3^Engineering Technology Research Center for Modern Animal Science and Novel Veterinary Pharmaceutic Development, Jiangsu Agri-animal Husbandry Vocational College, Taizhou, China; ^4^Department of Parasitology, Faculty of Veterinary Medicine, University of Firat, Elazig, Türkiye; ^5^National Research Center for Protozoan Diseases, Obihiro University of Agriculture and Veterinary Medicine, Obihiro, Japan

**Keywords:** BoSA1, cross-reaction, diagnosis, parasitemia, sandwich ELISA

## Abstract

Ovine babesiosis caused by *Babesia ovis* is an economically significant disease. Recently, a few *B. ovis*-specific proteins, including recombinant *B. ovis* secreted antigen-1 (rBoSA1), have been identified. Immunological analyses revealed that rBoSA1 resides within the cytoplasm of infected erythrocytes and exhibits robust antigenic properties for detecting anti-*B. ovis* antibodies. This protein is released into the bloodstream during the parasite’s development. It would be possible to diagnose active infections by detecting this secretory protein. For this purpose, a rBoSA1-specific polyclonal antibody-based sandwich ELISA was optimized in this study. Blood samples taken from the naturally (n: 100) and experimentally (n: 15) infected sheep were analyzed for the presence of native BoSA1. The results showed that native BoSA1 was detectable in 98% of naturally infected animals. There was a positive correlation between parasitemia level in microscopy and protein density in sandwich ELISA. Experimentally infected animals showed positive reactions from the first or second day of inoculations. However, experimental infections carried out by *Rhipicephalus bursa* ticks revealed the native BoSA1 was detectable from the 7^th^ day of tick attachment when the parasite began to be seen microscopically. Sandwich ELISA was sensitive enough to detect rBoSA1 protein at a 1.52 ng/ml concentration. Additionally, no serological cross-reactivity was observed between animals infected with various piroplasm species, including *Babesia bovis*, *B. bigemina*, *B. caballi, B. canis, B. gibsoni, Theileria equi*, and *T. annulata.* Taken collectively, the findings show that the rBoSA1-specific polyclonal antibody-based sandwich ELISA can be successfully used to diagnose clinical *B. ovis* infections in sheep at the early stage.

## Introduction

Ticks play a significant role in human and veterinary medicine due to their ability to transmit many protozoal, rickettsial, and viral diseases. Notably, protecting the small ruminants against tick infestations in the field is too hard because of their grazing behavior for long periods of the year. Ovine babesiosis is one of the most significant tick-borne protozoan diseases of livestock. Several epidemic and endemic cases caused by ovine babesiosis have been observed in Europe, the Middle East, North Africa, and some Asian countries (Yeruham et al., 1998; Fakhar et al., 2012; Ranjbar-Bahadori et al., 2012; Sevinc et al., 2013; Sevinc et al., 2018; Ceylan et al., 2021a; Ceylan et al., 2021b; Stevanovic et al., 2022).

Several *Babesia* species, namely *B. ovis, B. motasi, B. crassa, B. foliate*, and *B. taylori* have been identified in small ruminants, and cause ovine babesiosis. Recently, great attention has been paid to two new *Babesia* species in small ruminants, *Babesia* sp. Xinjiang in China (Yin et al., 1997; Bai et al., 2002) and *Babesia aktasi* n. sp. in Türkiye (Ozubek et al., 2023; Ulucesme et al., 2023), which presented distinct morphologies from other ovine *Babesia* species in thin blood smears. Within these species, *B. ovis* is responsible for clinical infections and has a wider distribution than other species (Uilenberg, 2006; Guan et al., 2009; Iqbal et al., 2011; Ozubek and Aktas, 2017; Ceylan et al., 2021b).

The most prominent clinical symptoms of *B. ovis* infections include fever, hemolytic anemia, and hemoglobinuria. In addition to these clinical symptoms; fatigue, loss of appetite, weight loss, and abortions may also occur in clinical cases. During the acute phase of infection, *B. ovis* merozoites proliferate and develop rapidly in erythrocytes. Then, they break down these erythrocytes and enter new erythrocytes. Some residual substances from the parasite and hemoglobin pass through the plasma. The presence of hemolytic anemia prevents the proper oxygenation of tissues, resulting in the onset of internal organ failures. Inevitably, when the level of parasitemia becomes elevated, it leads to a fatal outcome. Early and accurate diagnosis is the most critical part of disease control strategies. During the acute phase of the infection, the clinical signs may indicate the disease, and the parasites can be diagnosed by a specialist by examining the morphological characteristics of the parasite under a microscope. However, it is tough to diagnose the disease by microscopic and clinical examination methods in cases where the number of parasites is low (Yeruham et al., 1998; De Vos et al., 2000; Sevinc et al., 2013).

Serological diagnostic methods have generally been utilized to detect latent infections (Bose et al., 1995; Homer et al., 2000; Georges et al., 2001; Bock et al., 2004; Ceylan and Sevinc, 2020). Serological methods encompass both antibody-based techniques, which are mainly employed for gathering epidemiological information about diseases, and antigen-detection methods, which are utilized for diagnosing active infections. In cases where there is a low parasite count in the body, antigen detection methods can still identify the proteins secreted by the parasites. Consequently, these methods exhibit higher sensitivity compared to direct microscopy for the accurate diagnosis of ongoing infections (Montealegre et al., 1987; Chen et al., 2008; Luo et al., 2012).

Our group has recently described an immunoreactive protein named rBoSA1 (recombinant *B. ovis* secreted antigen-1) from *B. ovis*. We found that this protein had strong antigenic structures to detect anti-*B. ovis* antibodies. Additionally, we determined that the native BoSA1 protein was abundant in the cytoplasm of infected erythrocytes and corroborated that this protein was also detectable in the plasma of *B. ovis*-infected animals by western blot analysis (Sevinc et al., 2015). This unique secretory protein is predicted to be released into circulation from infected red blood cells due to intravascular hemolysis during the asexual development of the parasite. Active infections could be diagnosed by detecting this secretory protein *via* an antigen detection-based serologic method. Therefore, the present study aimed to develop a sandwich ELISA technique to detect native BoSA1 protein in serum and blood samples of sheep with active *B. ovis* infection.

## Materials and methods

### Blood samples, naturally and experimentally infected sheep

A hundred naturally infected sheep and 15 experimentally infected splenectomized lambs were included in the study. Natural infections were from the clinical cases detected in the central part of Türkiye (Sevinc et al., 2013). The experimental lambs underwent splenectomy utilizing established surgical techniques, as outlined in the previous study (Sevinc et al., 2007). Preimmune sera were collected from the lambs prior to initiating the experimental infection. Of the 15 experimental infections, 13 were performed by intravenous inoculation of *B. ovis*-infected blood (Sevinc et al., 2007; Sevinc et al., 2014). Imidocarb dipropionate (1.2 mg/kg) was used to treat animals that developed parasitemia during these infections. The remaining 2 experimental infections were carried out by unfed adult *Rhipicephalus bursa* infected with *B. ovis* in 5-6 months-old Anatolian Romanov lambs. The lambs were purchased from the Baskil district located in Elaziğ province of Türkiye, and housed in a closed pen at the Veterinary Medicine animal facility. The blood samples taken from the lambs before the experiment were subjected to microscopic, serologic, and molecular analyses, and it was confirmed that the lambs were free from blood parasites including *B. ovis* prior to being infested with infected ticks. The infected unfed *R. bursa* tick line continuing at the Parasitology Department of the Veterinary Faculty, Firat University was used for the experiment.

### Experimental infection in lambs by unfed adult *R. bursa* infected with *B. ovis*

To establish the timeframe for detecting native BoSA1 in the bloodstream, 90 and 100 adult unfed *R. bursa* infected with *B. ovis* were placed on the splenectomized lamb 1 and lamb 2, respectively (Erster et al., 2016). The infected ticks were fed on lambs until repletion in the plastic capsules glued to the backs of the animals (Almazán et al., 2018). After the infected ticks were attached, the lambs were periodically monitored for the progress of the infection. During the experiment, clinical findings and body temperature of each lamb were checked daily. Simultaneously, thin blood smears, EDTA blood samples and sera were collected for microscopic examination, PCR, and rBoSA1-specific polyclonal-antibody-based sandwich ELISA, respectively.

### Microscopic detection of *Babesia ovis*

Animals suspected to have the disease were first examined clinically, followed by a small incision from the ear tip of the animals. A few drops of blood were drawn from these incisions, and the thin blood smears were prepared. The prepared smears were stained with a 10% Giemsa solution for at least 30 min after fixing with methanol for 5 min. The level of parasitemia was determined by examining at least 20 microscope fields. Parasitemia levels were categorized according to relevant literature as follows: 1: Low parasitemia (0.1-0.3%), 2: Moderate parasitemia (0.4-0.9%), 3: High parasitemia (1-2.5%), and 4: Very high parasitemia (>2.5%) (Sevinc et al., 2013). Blood samples were collected from the jugular vein of the lambs using both anticoagulant-coated (EDTA) and non-anticoagulant vacuum tubes. The sera were separated by centrifugation and stored in a –20°C freezer until use. PCR analysis for detection of *B. ovis* DNA was performed as described previously (Aktas et al., 2005).

### Production of recombinant BoSA1 protein

Expression of rBoSA1 protein from *E. coli* DH5α cells and its purification were prepared as reported in the previous study (Sevinc et al., 2015).

### Mice and rabbit immunizations

To produce the capture and detection antibodies, twelve 6-week-old specific pathogen-free (SPF) ICR mice (CLEA, Japan) and one white rabbit weighing 2.5 kg were used to generate anti-rBoSA1 polyclonal antibodies. Mice were immunized by injecting 100 micrograms (µg) of purified rBoSA1 intraperitoneally after emulsifying with an equal volume of Freud’s complete adjuvant (Sigma-Aldrich, USA). At 14 and 28 days after the first immunization, the same amount of antigen was emulsified with Freud’s incomplete adjuvant (Sigma-Aldrich, USA), and the mice were given second and third immunizations by intraperitoneal injection again. In rabbit immunization, 1 mg of purified rBoSA1 antigen was administered subcutaneously to different points on the rabbit’s body, with emulsions made with Freud’s complete and incomplete adjuvants, as in mice. Rabbit immunizations were performed three times with an interval of two weeks, as in the mice. The whole blood of mice and rabbit immunized with antigen was collected 14 days after the last immunization, and immune sera were extracted by centrifugation. Anti-rBoSA1 polyclonal IgG antibodies in mice and rabbit sera were purified using the Econo-Pac protein A kit (BioRad Laboratories, USA) (Luo et al., 2012). Purified antibodies were stored in a –30°C deep freezer and then used in the sandwich ELISA method.

### Determination of detection limit of native BoSA1 protein by sandwich ELISA

To determine the minimum detection limit of the rBoSA1-specific polyclonal antibody-based sandwich ELISA, two-fold dilutions beginning from 200 µg/ml concentration of rBoSA1 were tested in sandwich ELISA.

### Sandwich ELISA applications

Sandwich ELISA assay was optimized by testing different dilutions of rabbit and mouse anti-rBoSA1 polyclonal IgGs and enzyme-labeled secondary antibody (HRP-conjugated goat-anti mouse IgG, Bethyl lab, USA). Twenty serum and blood samples collected from healthy lambs were used to establish a BoSA1-specific cut-off for sandwich ELISA. The cut-off value was calculated according to the formula of the mean optical density of the negative samples plus 2-fold the standard deviation. The ELISA microplate was first incubated at 4°C overnight with 100 μl of rabbit anti-rBoSA1 antibodies diluted with carbonate–bicarbonate buffer (0.05 M carbonate–bicarbonate buffer, pH 9.6) at a concentration of 2 μg/ml and then blocked in a 37°C incubator with phosphate-buffered saline (PBS) containing 5% skim milk powder (Sigma-Aldrich) solution for 1 h. After one washing with PBST (Phosphate buffered saline with Triton-X), 100 µl of the positive/negative control samples and infected animals’ serum/blood samples at dilution of 1/5 were added and incubated at 37°C for 1 h. Then, six washes were performed with PBST again. It was incubated with mouse anti-rBoSA1 antibodies diluted with 5% skim milk solution at a concentration of 2 μg/ml at 37°C for 1 h. Six more washes were repeated with PBST, and finally, the plate was incubated with the horseradish peroxidase (HRP)-conjugated anti-mouse IgG (Bethyl, USA) diluted with 5% skim milk solution at a ratio of 1/8000 at 37°C for 1 h. The wells were rewashed, and 100 µl ABTS (2,2’-azino-bis (3-ethylbenzothiazoline-6-sulphonic acid)) substrate (Sigma-Aldrich, USA) was added to each well, and then the plate was kept in the dark for 30 min. The intensity of the enzyme reaction was quantified using the 415 nm filter of the ELISA microplate reader (Rayto RT-2100C, China). Purified recombinant BoSA1 protein was used as a positive control in the sandwich ELISA.

### Control of serological cross-reactivity

To determine the specificity of the sandwich ELISA method, various piroplasm-positive serum samples, including *B. bovis*, *B. bigemina*, *B. caballi, B. canis, B. gibsoni, Theileria equi*, and *T. annulata* were tested in terms of native BoSA1 protein.

### Statistical analysis

The correlation between the parasitemia level of *B. ovis* infection and the optical density (OD value) of native BoSA1 protein in sandwich ELISA was statistically investigated using Spearman Correlation statistical analysis method. The SPSS 25 (IBM Corp. Released 2017. IBM SPSS Statistics for Windows, Version 25.0. Armonk, NY: IBM Corp.) statistical package program was adopted to analyze the data. *p*-Values were computed to determine the level of statistical significance. The significance level was indicated to be *p* < 0.05.

### Ethical statement

All applications performed on animals were conducted according to the conditions defined in the Selcuk University (Ethical Approval: 2016-35) and Firat University (Ethical Approval: 2021/12) Experimental Medicine Research and Application Center local ethical committee instructions. Rabbit and mice blood was collected under sedation by 5 mg/kg intramuscular xylazine hydrochloride (Rompun, Bayer) + 30-40 mg/kg ketamine hydrochloride (Ketalar, Pfizer) administration. After the blood was taken, the rabbit was euthanized by cervical decapitation, and the mice were euthanized by cervical dislocation.

## Results

### Detection limit of BoSA1 by sandwich ELISA

**Figure 1:**
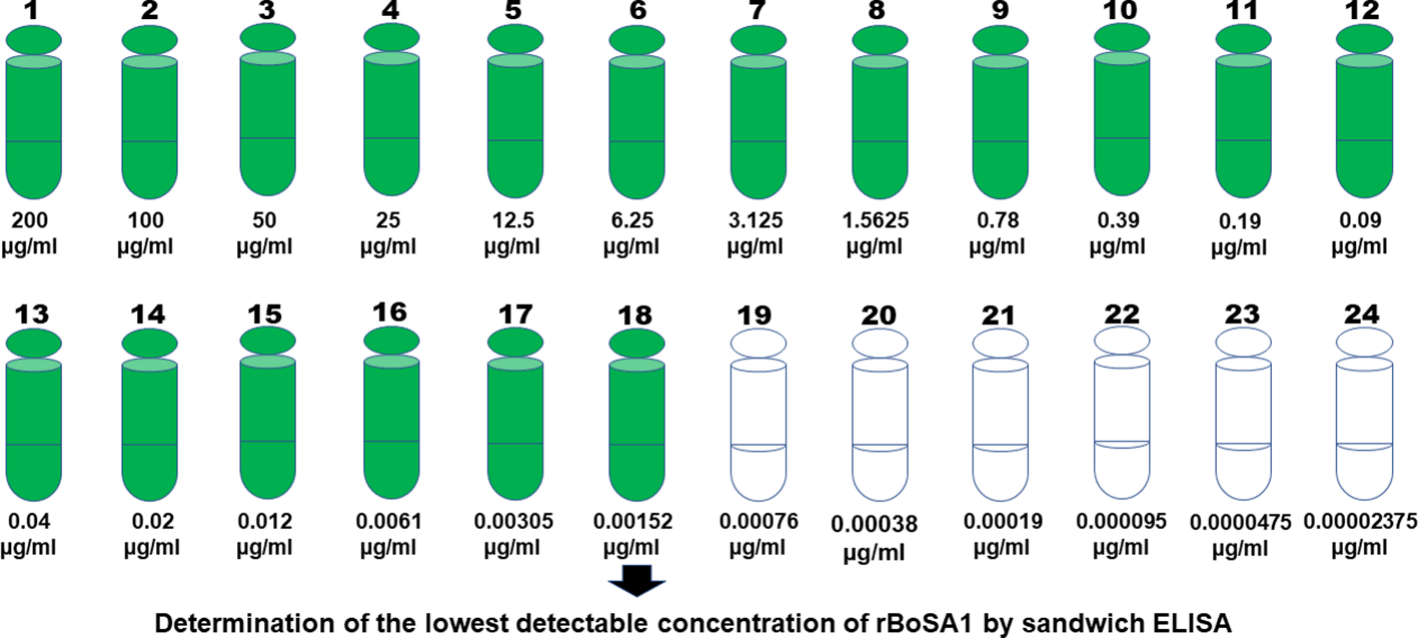
Twenty-four serial dilutions of rBoSA1 to determine the lowest detectable concentration by sandwich ELISA.

By analyzing 24 serial dilutions starting from 200 µg/ml concentration of rBoSA1, it was found that sandwich ELISA had a very high sensitivity to detect rBoSA1 proteins. The lowest detectable amount of rBoSA1 was 1.52 ng/ml. Detailed information is illustrated in **Figure 1**.

### The presence of native BoSA1 protein in the naturally infected sheep

**Figure 2:**
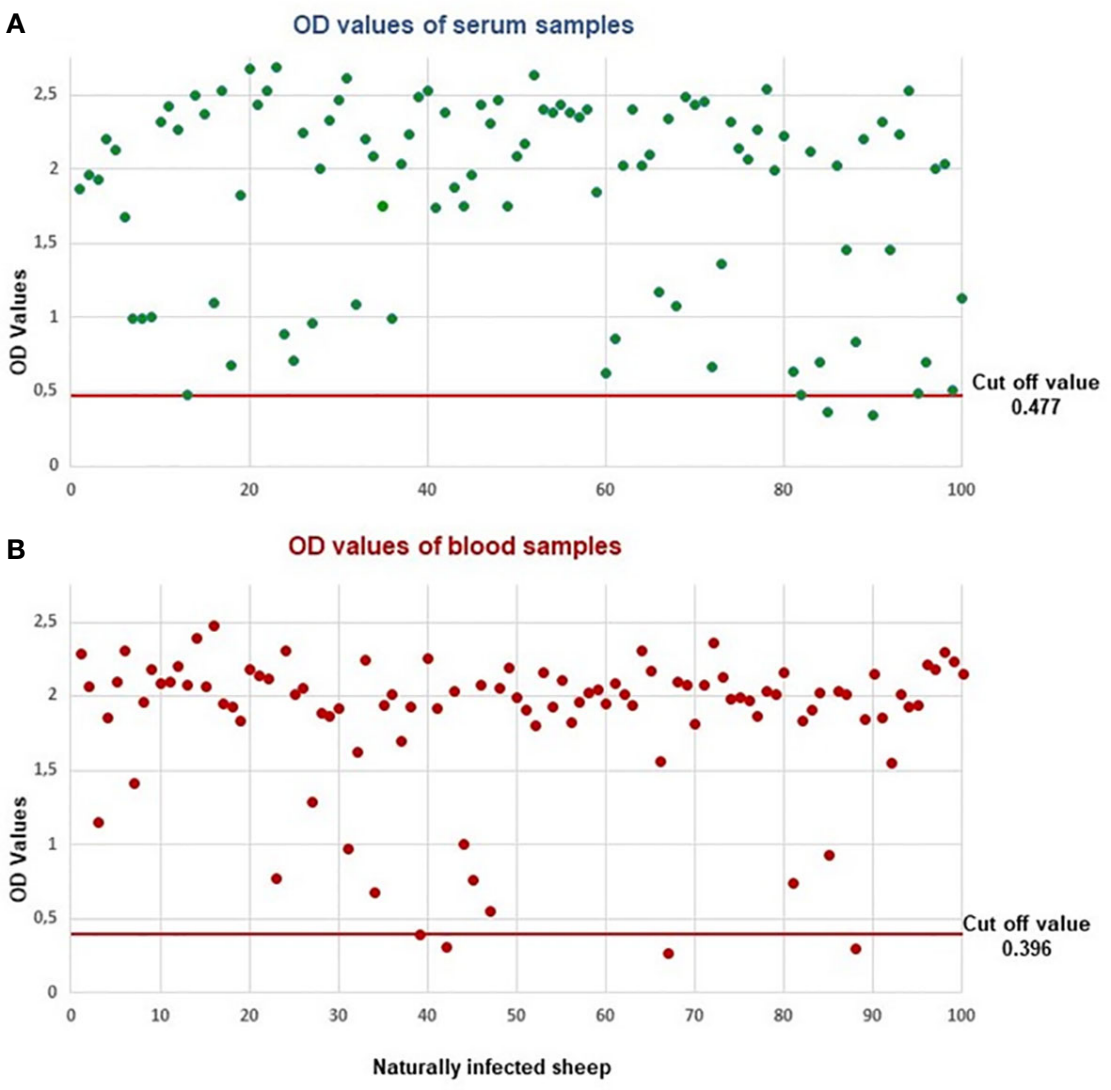
Sandwich ELISA results in the serum and blood samples of naturally infected sheep. **(A)** OD values of serum samples, **(B)** OD values of blood samples.

To determine the method’s sensitivity, the samples taken from 100 naturally infected sheep were examined for the presence of the native BoSA1 protein by sandwich ELISA. The cut-off calculated from healthy lambs was 0.477 for sera and 0.396 for blood. Circulating native BoSA1 protein was detected in 98 and 97 of the sera and blood samples, respectively. While OD values above 0.477 and 0.396 in infected sera and blood were detected respectively, it was well below these limits in the negative samples. Sandwich ELISA results of all serum and blood samples taken from naturally infected animals are illustrated in **Figure 2**.

### The dynamics of native BoSA1 protein in experimentally infected lambs

**Figure 3:**
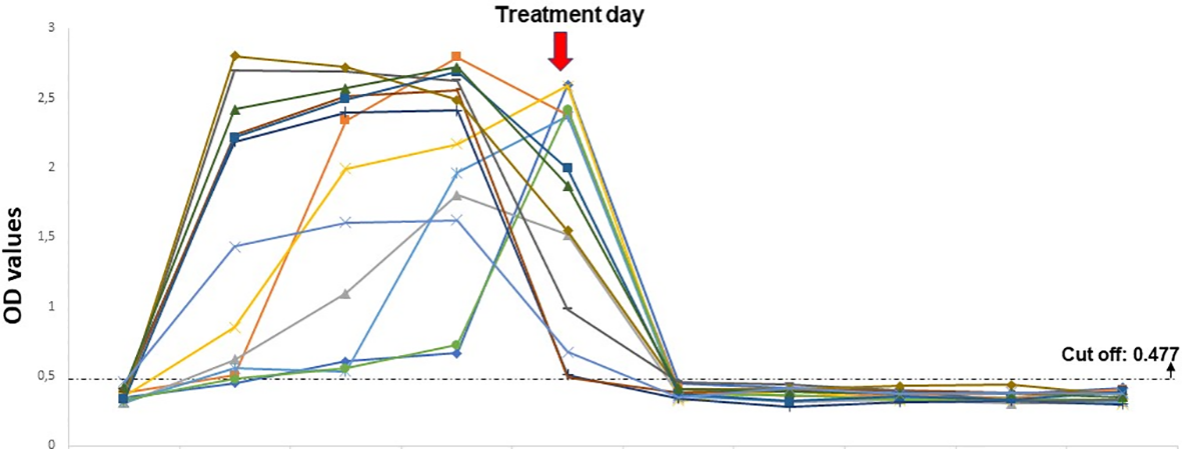
The dynamics of native BoSA1 protein in the experimental infections performed by *B ovis*-infected blood inoculation. The horizontal axis represents the pre- and post- inoculation days.

Serum samples from experimentally infected lambs showed positive reactions from the first and second days of the infected blood inoculations. Native BoSA1 protein was detected on the first day following the *B. ovis*-infected blood inoculation in twelve of the thirteen samples, and all lambs were positive on the second day of inoculation. The density of protein continued till the treatment time with high OD values. Native BoSA1 protein started to disappear on the first day of the drug (1.2 mg/kg imidocarb dipropionate) administration which mostly corresponded to the fourth day of experimental infection, and all samples were negative during the post-treatment period. Detailed information about the dynamics of native BoSA1 protein in the experimentally infected lambs by *B. ovis*-infected blood inoculation is given in **Figure 3**.

### The dynamics of native BoSA1 in the experimental infection by unfed adult *R. bursa* infected with *B. ovis*

This experiment was added to the study to find out the detection time of native BoSA1 protein in the blood of animals after tick attachment. The information to be obtained from this experiment will be extremely important for the diagnosis of infection under field conditions. Both splenectomized lambs infested unfed adult *R. bursa* infected with *B. ovis* developed severe clinical babesiosis, including anemia, hemoglobinuria, high fever, and high parasitemia of 7.1% (lamb 1) and 42.5% (lamb 2). Both lambs died 13 days post tick infestation, within 3-4 days from the onset of parasitemia (**Table 1**). In the infections carried out by *B. ovis*-infected *R. bursa* ticks, native BoSA1 could be detected from the 7^th^ day of tick attachment by rBoSA1-specific polyclonal-antibody-based sandwich ELISA. The time of appearance of the rBoSA1 protein nearly corresponded to the day when *B. ovis* merozoites began to appear microscopically in the blood. The detection of parasite DNA by PCR was 1-3 days before protein detection time. The OD value was above the cut-off on the seventh day and increased in the following days, except for an experimental infection with low parasitemia. Detailed information concerning sandwich ELISA results of experimental infections carried by *R. bursa* ticks is given in **Table 1**.

**Table 1:** Sandwich ELISA results of experimental infections carried by *R. bursa* ticks.

	Tick attachment													
		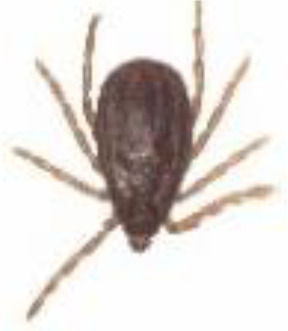			Days									
		1	2	3	4	5	6	7	8	9	10	11	12	13
**Lamb 1**	**Microscopy**	**-**	**-**	**-**	**-**	**-**	**-**	**-**	**-**	**+**	**+**	**+**	**+**	**Died**
	**PCR**	**-**	**-**	**-**	**-**	**-**	**-**	**+**	**+**	**+**	**+**	**+**	**+**	
	**S-ELISA**	**-**	**-**	**-**	**-**	**-**	**-**	**-**	**-**	**+**	**+**	**+**	**+**	
	**Parasitemia (%)**	**0**	**0**	**0**	**0**	**0**	**0**	**0**	**0**	**0.01**	**0.4**	**2.1**	**7.1**	
**Lamb 2**	**Microscopy**	**-**	**-**	**-**	**-**	**-**	**-**	**-**	**-**	**-**	**+**	**+**	**+**	**Died**
	**PCR**	**-**	**-**	**-**	**-**	**-**	**-**	**-**	**+**	**+**	**+**	**+**	**+**	
	**S-ELISA**	**-**	**-**	**-**	**-**	**-**	**-**	**-**	**-**	**+**	**+**	**+**	**+**	
	**Parasitemia (%)**	**0**	**0**	**0**	**0**	**0**	**0**	**0**	**0**	**0**	**0.01**	**11.7**	**42.5**	

### Cross-reactivity

There was no cross-reactivity against polyclonal anti-rBoSA1 antibodies in the sera from various animals infected with piroplasm species, including *B. bovis*, *B. bigemina*, *B. caballi, B. canis, B. gibsoni, T. equi*, and *T. annulata.*

### Positive correlation between parasitemia level and OD value

**Figure 4:**
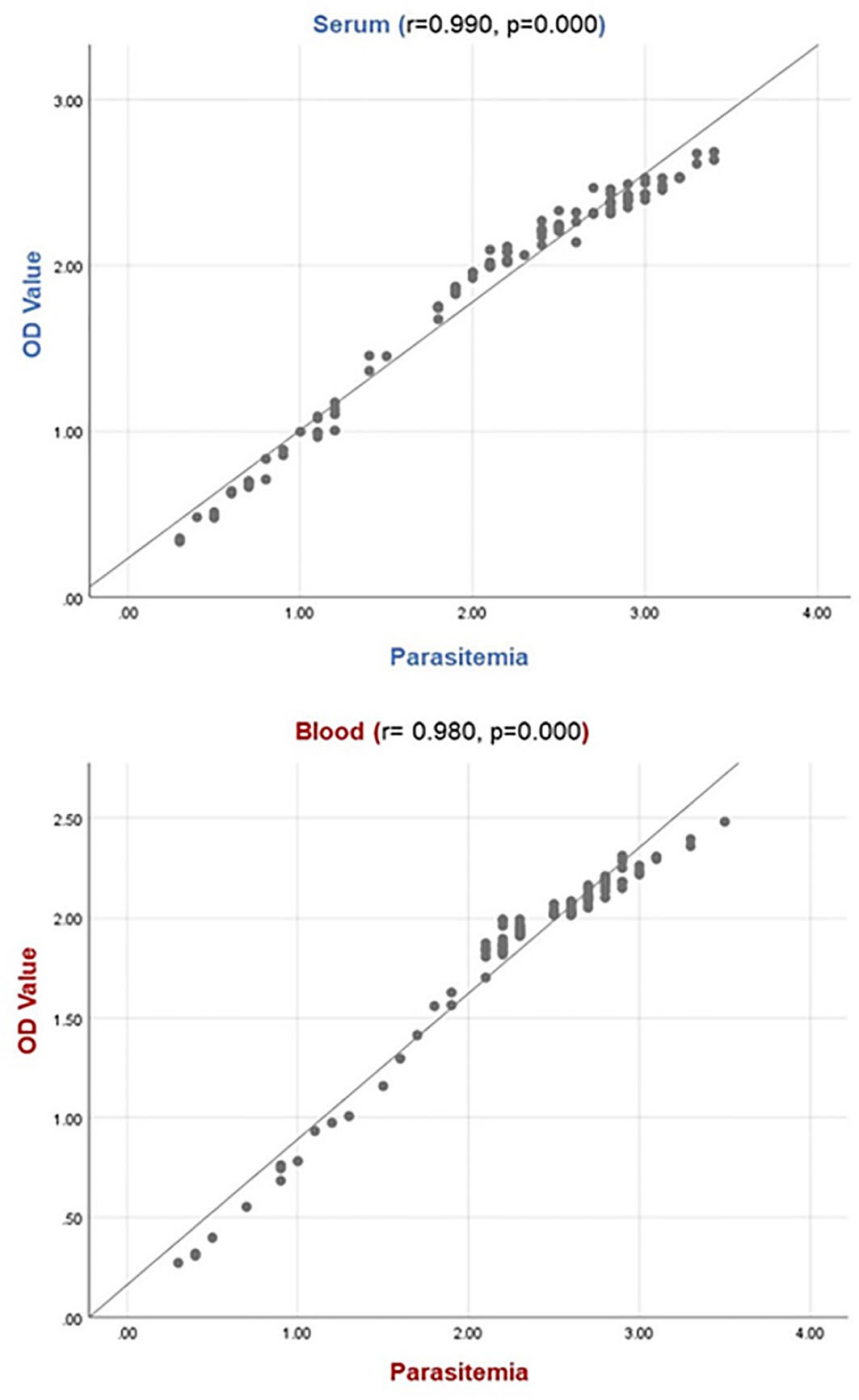
Relation between OD value and parasitemia level. The relationship between parasite load and OD value indicates there is a positive correlation for serum samples (r: 0.990, *p*: 0.000) and blood samples (r: 0.980, *p*: 0.000). For the parasitemia line: 1, 2, 3, and 4 indicate the low (0.1-0.3%), moderate (0.4-0.9%), high (1-2.5%) and very high (>2.5%) parasitemia levels, respectively.

A statistically significant relationship was determined between parasitemia levels and OD values of naturally infected animals’ serum and blood samples. It was observed that the OD value increased as the parasitemia level increased, and the OD value decreased as the parasitemia level decreased. The graphs obtained from Spearman correlation analysis are shown in **Figure 4**.

## Discussion

Ovine babesiosis is usually associated with mortality and morbidity in small ruminants. The incubation period following tick attachment is around two weeks. At the end of this period, the body temperature increases up to 40-42°C. Loss of appetite and depression are observed in animals. The respiration and pulse rates elevate because of hemolytic anemia due to the breakdown of red blood cells, and hemoglobinemia, hemoglobinuria, and jaundice are observed. Manifestations including the presence of bloody stools, muscle tremors, and leg paralysis can be observed as symptoms of the infection. In cases of acute babesiosis, if left untreated, the disease can lead to fatality, with the primary cause of death typically being anoxia resulting from anemia (Yeruham et al., 1998). Erythrocytes are the first and only settlement area of the *Babesia* species in vertebrate hosts. *Babesia ovis* merozoites develop by dividing into two or more in erythrocytes, break down the erythrocytes, and enter other erythrocytes to be able to continue asexual development. As a result of a series of asexual reproduction, most of the erythrocytes become infected, and some residual substances from the parasite pass through the plasma (Yeruham et al., 1998; Uilenberg, 2006; Sevinc et al., 2013).

Secretory proteins found in the parasite structures are the richest resources for new therapeutic drug targets, diagnostic antigens, and vaccine candidates (Bonin-Debs et al., 2004; Ceylan et al., 2022). Parasite proteins secreted into the host cell play a vital role in modifying the host cell and provide the interaction between the host immune system and the apicomplexan parasites including intracellular protozoans such as *Toxoplasma*, *Plasmodium* and *Theileria* (Ravindran and Boothroyd, 2008). During the asexual development of *Babesia* species in erythrocytes, parasite proteins are released and can be found in both the cytoplasm of erythrocytes and blood plasma. The majority of these parasite proteins are capable of eliciting an immune response. It means that they stimulate the immune system by activating cellular and humoral defense factors when secreted. Therefore, immunoreactive proteins are the target molecules used to diagnose diseases (Sahagun Ruiz et al., 2000; Kumar et al., 2002; Huang et al., 2006; Terkawi et al., 2007; Goo et al., 2008; Ramos et al., 2009; Ooka et al., 2011; Ceylan et al., 2022).

Successful treatment of *B. ovis* infection depends on an early and accurate diagnosis. During an acute infection, the disease is diagnosed by observing babesiosis-related clinical symptoms and analyzing the morphological structures of the merozoites in the erythrocytes. However, there are some diseases confusing with babesiosis because of the similarity of clinical signs, such as leptospirosis, anaplasmosis, and copper poisoning. Besides, the parasites are not demonstrable in the blood under a microscope, especially in the case of subclinical infections. Various serological antibody detection methods have been used to detect latent or subclinical infections with low parasitemia (Bose et al., 1995; Georges et al., 2001; Bock et al., 2004). The indirect fluorescence antibody (IFA) test is a sensitive method for serological diagnosis of babesiosis (Homer et al., 2000). Factors such as the occurrence of cross-reactions between different species (Bose et al., 1995), the lack of automated testing procedures, semi-quantitative evaluation of results, and the need for a specialist and fluorescence microscope to evaluate the results are among the disadvantages of the IFA test. Various ELISA methods have been used to detect specific antibodies against *Babesia* species such as *B. bovis*, *B. bigemina*, *B. divergens*, *B. caballi*, *B. canis*, *B. gibsoni*, *B. microti*, and *Babesia* sp. Xinjiang (Bose et al., 1990; Kappmeyer et al., 1999; Ikadai et al., 2000; Fukumoto et al., 2001; Boonchit et al., 2002; Goff et al., 2003; Goff et al., 2006; Goff et al., 2008; Niu et al., 2016; Chung et al., 2017; Ortiz et al., 2018). Recently, sandwich ELISA techniques based on antigen detection have been developed for the diagnosis of blood parasite infections (De Arruda et al., 2004; Dondorp et al., 2005; Chen et al., 2008), including *B. bovis* and *B. microti* infections (Montealegre et al., 1987; Luo et al., 2012; Thekkiniath et al., 2018). The literature review showed that there is no commercial diagnostic test based on antigen detection in the diagnosis of ovine babesiosis caused by *B. ovis*. Our group has recently identified a secretory protein named recombinant *B. ovis*-secreted antigen 1 (rBoSA1) of *B. ovis*. It was characterized as an immunoreactive protein that can be used to develop serological methods for diagnosing babesiosis in sheep, and the immunofluorescence assay results revealed that native BoSA1 protein located on the surface and inside of *B. ovis* merozoites, and the parasite intensely secreted the protein to the cytoplasm of the infected erythrocytes (Sevinc et al., 2015). These circulating *B. ovis* proteins can be detected earlier than the formation of specific antibodies in the blood by a method based on antigen detection, and they can serve as biomarkers in the early diagnosis of *B. ovis* infection. This study was carried out to detect native BoSA1 protein in the blood and serum samples of the infected animals using the sandwich ELISA technique and eventually diagnose babesiosis in sheep during active infection. The sandwich ELISA technique was applied using polyclonal antibodies specific for recombinant BoSA1 protein. The polyclonal anti-rBoSA1 antibodies detected the circulating native BoSA1 protein with high sensitivity in 98% of the animals, which were positive for *B. ovis* infection by microscopy and PCR analysis (**Figure 2**). In ELISA, only two serum samples from naturally infected animals were negative. There was a statistically positive correlation between the OD values of these samples and parasitemia levels. Our previous study (Sevinc et al., 2015) showed that 68.42% of naturally infected sheep were positive for *B. ovis-*specific antibodies during active infections. It is inferred that the sandwich ELISA technique is more sensitive than indirect ELISA in detecting clinical infections. Antibodies, the basic elements of humoral defense, are usually seen in circulation toward the last stages of acute infections and continue to exist in the body for several years. Therefore, the detection of the parasite-specific antibodies in circulation indicates that the parasite infects the host; however, it does not provide any information about the infection time. On the other hand, the detection of a specific parasite protein in blood indicates that the parasite-originated infection still exists in the body. Hence, antigen detection-based methods should be used to diagnose active infections, rather than antibody detection-based serological methods.

Additionally, the sera from 13 lambs experimentally infected with *B. ovis*-infected blood showed a positive reaction, at OD values above 0.477, from the first or second day of experimental infections. The OD values of native BoSA1 increased progressively in the acute period. At this period, *B. ovis* merozoites asexually propagated quickly (**Figure 3**). Then, OD values decreased significantly on the 1^st^ post-treatment day following imidocarb dipropionate (1.2 mg/kg) administration and decreased below the cut-off value on the 2^nd^ post-treatment day (**Figure 3**). When these results were compared with the previous studies (Sevinc et al., 2007; Sevinc et al., 2014; Sevinc et al., 2015) where *B. ovis*-specific antibodies could be detected on the 7^th^ or 8^th^ days of experimental infections by blood inoculation, sandwich ELISA is more sensitive than indirect ELISA for early diagnosis of clinical infections. On the other hand, *B. ovis*-infected *R. bursa* ticks-induced experimental infections mimicking natural infection, native BoSA1 was detected in blood serum from the 7^th^ day of tick attachment. The detection time of native BoSA1 protein in sera and the appearance of *B. ovis* merozoites in Giemsa-stained thin blood smears were compared. Accordingly, while the lamb1 was found positive for the presence of the native BoSA1 protein on the day of microscopically detection time of merozoites, lamb2 showed positivity in Sandwich ELISA one day before the detection of *B. ovis* merozoites (**Table 1**). The detection of parasite DNA by PCR was one or two days before protein detection. All performed experiments suggest that native BoSA1 is a promising indicator for detecting early-stage of *B. ovis* infections, and the new sandwich ELISA technique described in the study is a much more sensitive method to diagnose active *B. ovis* infections when compared to antibody detection assays.

Serial dilutions of rBoSA1 were tested to determine the lowest detection limit of sandwich ELISA. As a result of the test, sandwich ELISA was highly sensitive to detect as little as 1.52 ng of rBoSA1 protein in 1 ml solution (**Figure 1**). In addition, there was a significantly positive correlation between OD values and parasitemia levels (r: 0.980, *p*: 0.000 for blood, r: 0.990, *p*: 0.000 for serum) in the present study (**Figure 4**). Similar findings were reported by Joshi et al. (2004) in *Plasmodium vivax* infections of humans, and by Luo et al. (2012) in *B. microti* infections of hamsters. Luo et al. (2012) revealed that circulating *Babesia microti*-secreted antigen 1 (BmSA1) overlaps with the parasitemia profile during active infections in a hamster model. It is concluded that rBoSA1-specific polyclonal antibody-based sandwich ELISA may provide information about the severity of acute infections and *B. ovis* burden in circulation in the clinical evaluation of ovine babesiosis.

The major disadvantage of serological tests is cross-reactions (Jiang et al., 2021). In order to reduce the possibility of cross-reaction, antigenic fractions of the parasite in the host circulation should be used in serological methods (Petray et al., 1992; Chen et al., 2008). No cross-reactivity against polyclonal anti-rBoSA1 antibodies in the sera of different animal species which were positive for the apicomplexan parasites, including *B. bovis*, *B. bigemina*, *B. caballi, B. canis, B. gibsoni, T. equi*, and *T. annulata* was detected in this study. This result indicated that the native BoSA1 protein has no common antigenic determinants with the aforementioned piroplasm species, and it is a strong immunoreactive protein specific for *B. ovis*. The findings indicate that this new sandwich ELISA technique can successfully diagnose ovine babesiosis caused by *B. ovis* during active infection without cross-reaction.

In conclusion, this is the first study on the sandwich ELISA technique for diagnosing *B. ovis* infection in its active phase. By sandwich ELISA, native BoSA1 protein was captured in almost all samples examined with high sensitivity and specificity in sheep. When the ELISA results of the positive and negative samples tested in this study are evaluated, it is seen that the sensitivity is 98.26% (113 of a total of 115 naturally and experimentally infected samples) and the specificity is 100% (all of the 20 negative samples examined for cut-off calculation). Early diagnosis is crucial to control *B. ovis* infection, which is especially common in tropical and subtropical countries and causes deaths in sheep (Sevinc et al., 2018; Ceylan and Sevinc, 2020). The application of the rBoSA1-specific polyclonal antibody-based sandwich ELISA technique yielded noteworthy findings concerning the timing of native BoSA1 protein detection in experimental infections induced through infected blood inoculation and *R. bursa* ticks infected with *B. ovis*. The study findings demonstrate that the rBoSA1-specific polyclonal antibody-based sandwich ELISA method can be employed with success and reliability for the early detection of acute *B. ovis* infections. Furthermore, it has the potential to be utilized in the evaluation of therapeutic drug effectiveness.

## Data availability statement

The datasets presented in this study can be found in online repositories. The names of the repository/repositories and accession number(s) can be found in the article/supplementary material.

## Ethics statement

The animal studies were approved by Selcuk University (Ethical Approval: 2016-35) and Firat University (Ethical Approval: 2021/12) Experimental Medicine Research and Application Center local ethical committee. The studies were conducted in accordance with the local legislation and institutional requirements. Written informed consent was obtained from the owners for the participation of their animals in this study.

## Author contributions

FS, MZ, SC, and XX designed the study. FS, MZ, SC, OC, MU, SO, and MA performed the experiments and analyzed the results. FS and OC wrote the draft of the manuscript. All authors read and approved the final manuscript.
